# Case report: A case of holocarboxylase synthetase deficiency with respiratory tract as the initial symptom

**DOI:** 10.3389/fgene.2024.1439343

**Published:** 2024-11-20

**Authors:** Haiying Zou, Li Yang, Renlong Zhang, Yao Qin

**Affiliations:** Department of Endocrinology, Genetics and Metabolism, Jiangxi Provincial Children’s Hospital, Nanchang, Jiangxi, China

**Keywords:** holocarboxylase synthetase deficiency (HLCSD), biotinidase deficiency, HLCS gene, mutation, biotin

## Abstract

**Introduction:**

Holocarboxylase synthetase deficiency (HLCSD) is a rare autosomal recessive genetic disorder caused by mutations in the holocarboxylase synthetase (HLCS) gene, which affects multiple systems. Common clinical manifestations include metabolic acidosis, rash, feeding difficulties, and growth retardation, with predominant involvement of the nervous system, skin, and hair. However, respiratory symptoms as the initial manifestation are relatively rare.

**Case Presentation:**

We report the case of a 1 year and 4-month-old Chinese male patient who presented with a 2-day history of cough, followed by half a day of wheezing and shortness of breath. Despite supportive treatment with antibiotics upon admission, the infant continued to experience rapid and deep breathing accompanied by groaning, and obvious wheezing. Blood gas analysis revealed metabolic acidosis that was difficult to correct. Blood tandem mass spectrometry showed elevations in C50H, C3, C4OH, and urine organic acid analysis revealed elevations in lactate, 3-hydroxybutyric acid, 3-hydroxyisovaleric acid, acetoacetic acid, 3-methylcrotonylglycine, and methylcitric acid. Genetic testing revealed two variants in the HLCS gene in the infant: NM_001352514: exon6: c.1088T>A: p.V363D variant and exon11: c.2434C>T: p.R812* heterozygous variant, resulting in HLCSD. Ultimately, the diagnosis of HLCSD was established, and oral biotin treatment achieved good clinical efficacy.

**Conclusion:**

This article summarizes the clinical data of a case of HLCSD in an infant, primarily presenting with respiratory symptoms. It provides a comprehensive summary of the etiology, diagnosis, and treatment, offering insights for the diagnosis of rare diseases by clinical physicians.

## 1 Introduction

Holocarboxylase synthetase (HLCS) deficiency (HLCSD), a rare autosomal recessive metabolic disorder, disrupts the binding of biotin to four specific apocarboxylases: pyruvate carboxylase, acetyl-CoA carboxylase, propionyl-CoA carboxylase, and methylcrotonyl-CoA carboxylase (L et al., 1985). HLCSD (OMIM#253270), also referred to as early-onset multiple carboxylase deficiency (MCD), typically becomes apparent shortly after birth. In contrast, biotinidase deficiency (BTD) is the form of MCD that presents later in life (Canda et al., 2020). Common manifestations of HLCSD include eczema, alopecia, lactic acidosis, hyperammonemia, seizures, and developmental delay (Tammachote et al., 2010). Treatment with biotin has demonstrated effective results (Wolf, 2022). Individuals who do not receive biotin therapy may experience psychomotor delay and severe metabolic acidosis, which can lead to unconsciousness or even fatality. It is important to note that HLCSD with respiratory tract involvement as the initial symptom is a rare occurrence.

HLCS deficiency may impacts respiratory health primarily through three interconnected mechanisms. Firstly, energy metabolism dysfunction occurs due to carboxylases, which are essential for energy generation, being impaired (Ferreira et al., 2021). This leads to an inadequate energy supply that can affect the normal function of the lungs and other organs (Chen et al., 2023). Secondly, HLCS deficiency can cause an accumulation of organic acids, potentially leading to metabolic acidosis (van der Knaap et al., 1998). This condition disrupts respiratory function as the body attempts to correct the blood pH imbalance by increasing the respiratory rate. Thirdly, biotin’s critical role in cell growth and DNA repair means its deficiency can compromise the immune system, increasing susceptibility to respiratory infections. Together, these effects highlight the complex ways in which HLCS deficiency can compromise respiratory health (Sullivan et al., 2023).

The mutation spectrum of the HLCS gene is associated with various clinical phenotypes, making molecular genetic analysis essential for a definitive diagnosis. These mutations can include missense mutations, deletions, as well as some total deletions and complex rearrangements (Yang et al., 2001). Recently, there have been reports of an increasing number of novel pathogenic variants (Wu et al., 2020). Interestingly, two mutations appear to be commonly observed in Chinese populations with HLCS deficiency (Zheng et al., 2020; Ling et al., 2023; Li et al., 2023). Furthermore, a study identified a novel heterozygous variant, c.996G > C (p.Gln332His), and a paracentric inversion on chromosome 21 (Quinonez et al., 2017). Additionally, there were five cases of HLCS deficiency with varying initial presentations and phenotypes (Donti et al., 2016). The sequencing of the HLCS gene helps confirm diagnoses and offer genetic counseling, while early treatment with pharmacological doses of oral biotin can prevent further decompensation in most cases before irreversible neurological damage occurs (Shao et al., 2022). In this report, we present a case of HCSD with respiratory tract symptoms as the initial manifestation, accompanied by metabolic acidosis and absence of a rash. The diagnosis of HCSD was established through molecular analysis.

## 2 Case description

**FIGURE 1:**
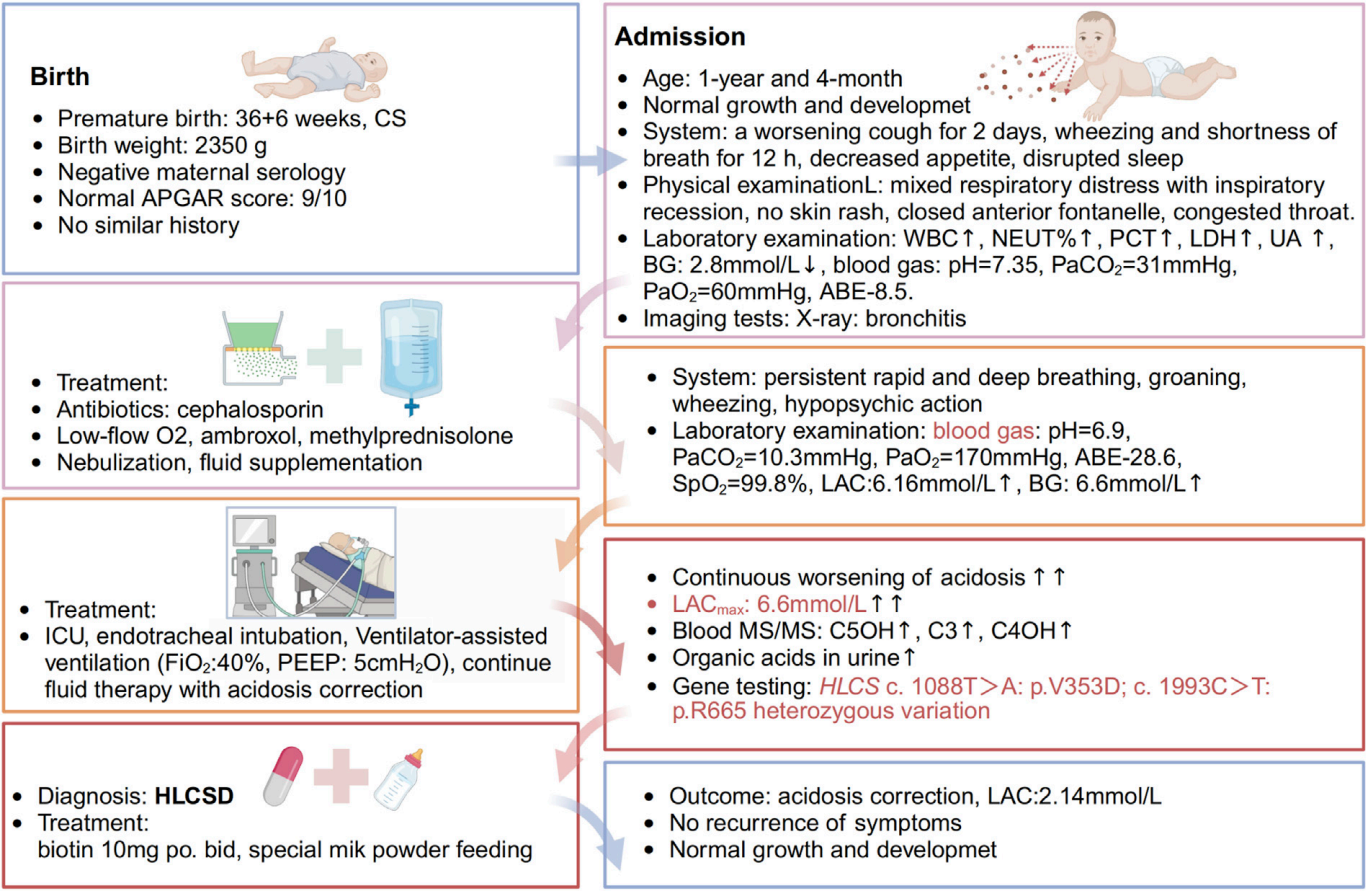
Timeline of events. A 1-year and 4-month-old Chinese male infant presented with a worsening cough for 2 days, accompanied by wheezing and shortness of breath for half a day. The patient initially experienced episodes of wet coughing, starting 2 days ago, with mild symptoms, and without fever, convulsions, or rash. For 6 h before admission, the patient also had a runny nose and one episode of non-projectile vomiting with moderate stomach contents. The patient underwent oral administration of Chinese patent medicines “Qing Kai Ling” and “Fei Li Kou” at a local hospital; however, no discernible improvement was observed. The cough worsened, accompanied by rapid breathing and wheezing, and the patient’s mental status and appetite gradually deteriorated. As a result, the decision was made to seek treatment at our hospital. The patient has exhibited decreased appetite, disrupted sleep, daily yellow soft stools, pale yellow urine, and decreased urine output. There is no known history of recurrent respiratory distress in the patient, who is the second infant in the family. The patient was delivered by Cesarean section at 36 weeks and 6 days of gestation, with a birth weight of 2.35 kg. The patient’s growth and development have been observed to be similar to other infant of the same age. Both parents of the infant are healthy and non-consanguineous, and there is no record of any similar illness within the family. The patient was afebrile, with no diarrhea. He demonstrates normal development and clear consciousness, but presents with a weak mental state and rapid breathing. There is no presence of skin rash. The anterior fontanelle is closed. The patient has red lips, dry oral mucosa, congested throat, and no enlarged tonsils or purulent discharge. Mixed respiratory distress with inspiratory recession is observed. Coarse respiratory sounds and audible wheezing are noted in both lungs. The heart rate is recorded at 145 beats per minute with a regular rhythm and strong heart sounds, and no murmurs are present. The abdomen is soft with no masses, tenderness, or rebound tenderness, and the subcutaneous fat thickness is measured at 8 mm. No other abnormalities are found during the physical examination. The patient’s admission laboratory examination revealed several abnormalities. There was an increase in white blood cells and neutrophil percentage, as well as elevated levels of procalcitonin, lactate dehydrogenase, and uric acid. Additionally, the patient had hypoglycemia with a blood glucose level of 2.8 mmol/L. The blood gas analysis showed a pH of 7.35, PaCO_2_ = 31 mmHg, PaO_2_ = 60 mmHg, and a base deficit of −8.5. Mild decreases were observed in the levels of IgG and complement C3. Coagulation, erythrocyte sedimentation rate, routine urine and stool tests, and pathogen testing yielded normal results. Liver function, electrolytes, and blood lipids were also within the normal range. Allergen testing came back negative. The chest X-ray showed evidence of bronchitis, while the abdominal color Doppler ultrasound did not reveal any abnormalities in the liver, gallbladder, or spleen. Echocardiography showed no apparent abnormalities in the heart. Brain magnetic resonance imaging demonstrated patchy T2 flair hyperintensities in the subcortical white matter of the bilateral lateral ventricular posterior corner and frontal lobe, suggesting possible delayed myelination. Furthermore, there was local enlargement in the left temporal horn and a smaller volume of the left hippocampus compared to the right side. Initially, the patient received cephalosporin to treat the infection, along with low-flow oxygen, ambroxol to aid expectoration, and methylprednisolone to reduce airway hyperreactivity. Treatment also included budesonide, salbutamol, and ipratropium bromide nebulization, as well as fluid supplementation. Despite these interventions, the infant exhibited persistent rapid and deep breathing with groaning, obvious wheezing, and severely impaired mental response. As a result, the infant was transferred to the intensive care unit for further evaluation.

A 17-month-old Chinese male infant presented with a worsening cough and shortness of breath over 2 days, alongside wheezing. Symptoms started with a wet cough, progressing to rapid breathing and deteriorating mental state. Treatments with traditional Chinese medicines “Qing Kai Ling” and “Fei Li Kou” showed no improvement. On admission, he had decreased appetite and was dehydrated but had no fever. Laboratory findings showed elevated white blood cells and neutrophils, hypoglycemia, and mild acidosis. A chest X-ray confirmed bronchitis. Further details are provided in Figure 1. Moreover, Urine analysis confirmed increased levels of lactate, 3-hydroxybutyric acid, 3-hydroxyisovaleric acid, acetoacetic acid, and 3-methylglutaconic acid, and decreased levels of methylmalonic acid. Table 1 presents the biochemical parameters measured at relevant time points. With the family’s consent, comprehensive whole-exome genetic testing was performed, identifying compound heterozygous variants in the HLCS gene: NM_001352514: exon6: c.1088T>A: p.V363D and exon11: c.2434C>T: p.R812*, which are considered pathogenic mutations. The diagnosis of HCSD was established, and treatment included the administration of biotin (10 mg, twice daily), and specialized formula feeding. Following treatment, the acidosis resolved, and the lactate level decreased to 2.4 mmol/L. The patient’s condition improved, and they were subsequently discharged.

**TABLE 1:** The biochemical parameters measured at relevant time points.

Timing	Sample	Index	Result	Reference range
First detection	Blood	WBC (g/L)	20.27	(5.5, 13.6)
		NEUT (%)	75.6	(13, 54)
		RBC (*10 ^ 12/L)	4.88	(4.1, 5.5)
		PCT (ng/mL)	11.16	≤0.5
		LDH (U/l)	571	(100, 240)
		Glucose (mmol/L)	2.8	(3.89, 6.11)
		IgG (g/L)	3.22	(3.49, 11.39)
		Complement C3 (g/L)	0.82	(0.88, 1.6)
	Blood gas	pH	7.35	(7.35, 7.45)
		PaCO2 (mmHg)	31	(35, 45)
		PaO2 (mmHg)	60	(80, 100)
		ABE (mmol/L)	−8.5	(−3, 3)
	Urine	Uric Acid (μmol/L)	759.61	(123, 430)
Follow-up detection	Blood gas	pH	6.931	(7.35, 7.45)
		PaCO2 (mmHg)	10.3	(35, 45)
		PaO2 (mmHg)	170	(80, 100)
		ABE (mmol/L)	−28.6	(−3, 3)
	Blood	Glucose (mmol/L)	6.6	(3.89, 6.11)
		Lactic acid (mmol/L)	6.6	(0.44, 1.78)
		C3 (μM)	14.375	(0.65–6.04)
		C4OH (μM)	0.579	(0.03–0.34)
		C5OH (μM)	13.633	(0.1–0.61)
		C5OH/C0	1.617	(0–0.03)
		C5OH/C3	0.948	(0.05–0.47)
		C5OH/C8	258.758	(1.5–13.5)
Follow-up detection after 2 months of treatment	Blood	C3 (μM)	5.1	(0.65–6.04)
		C4OH (μM)	0.156	(0.03–0.34)
		C5OH (μM)	5.876	(0.1–0.61)
		C5OH/C0	0.09	(0–0.03)
		C5OH/C3	1.152	(0.05–0.47)
		C5OH/C8	90.363	(1.5–13.5)

### 2.1 Genetic analysis

**FIGURE 2:**
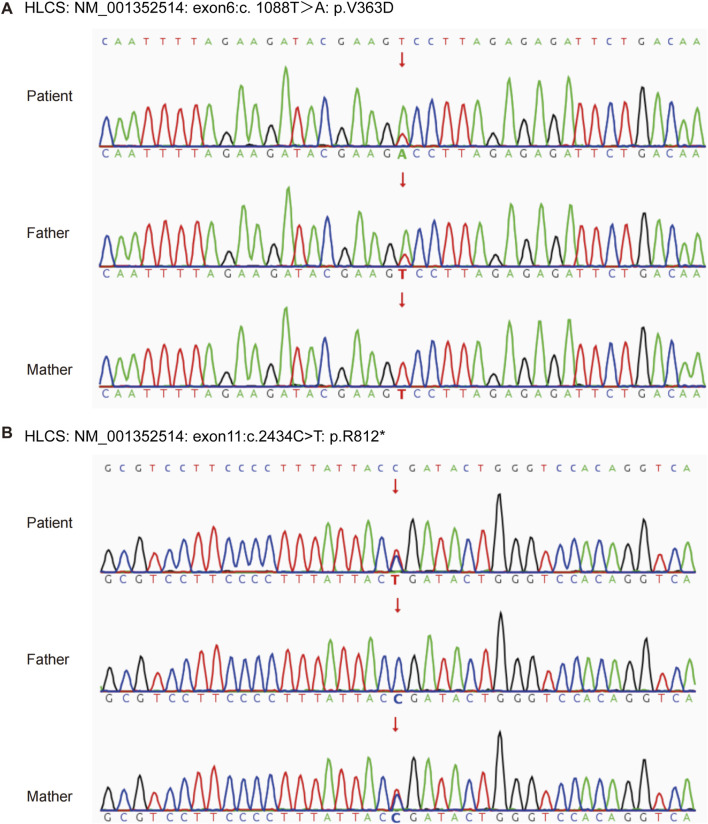
SNV and InDel site maps. **(A)** HLCS: NM_001352514: exon6: c.1088T>A: p.V363D. **(B)** HLCS: NM_001352514: exon11: c.2434C>T: p.R812*. * Indicates that the mutation is not included in the database.

The patient and his non-consanguineous parents underwent comprehensive physical examinations and genetic testing. Whole-exome sequencing (WES) was conducted using the Verita Trekker^®^ variant detection system and the Enliven^®^ variant annotation and interpretation system developed by Berry Gene. PCR and capillary electrophoresis were employed for a comprehensive analysis of the WES results pertaining to dynamic mutations. Table 2 presents the single nucleotide variants (SNVs) and Insertions/deletions (InDel) findings. The patient’s sample revealed two variants of the HLCS gene: a pathogenic variant, HLCS: NM_001352514: exon6: c.1088T>A: p.V363D, and another pathogenic variant, HLCS: NM_001352514: exon11: c.2434C>T: p.R812*, which is associated with HLCSD (OMIM:253270). Following guidelines from the American College of Medical Genetics and Genomics (ACMG) (Richards et al., 2015), recommendations from the ClinGen Sequence Variant Interpretation (SVI) Expert Panel (Abou Tayoun et al., 2018; Biesecker and Harrison, 2018; Ghosh et al., 2018), and screening for phenotype-related gene variants in databases like HPO, OMIM, and GHR, the c.1088T>A mutation of the HLCS gene is categorized as pathogenic, and the c.1993C>T mutation is also deemed to be pathogenic. Figure 2 displays the SNV and InDel results for the patient and parents, illustrating that the patient inherited a heterozygous c.1088T>A mutation in the HLCS gene from the father and a heterozygous c.2434C>T mutation from the mother.

**TABLE 2:** SNV and InDel test results.

Gene	Mutation site	Gene region	HGVS	Mutation type	Heterozygosity	Variation grade	Disease and hereditary mode
*HLCS*	chr21:36930342-36930342	exon6	NM_001352514:c. 1088T>A: p. V363D	missense_variant	Patient: heterozygosis Father: heterozygosis Mother: wild type	Pathogenic	HLCSD, AR
*HLCS*	chr21:36756558-36756558	exon11	NM_001352514:c.2434C>T: p.R812*	stop_ gained	Patient: heterozygosis Father: wild type Mother: heterozygosis	Pathogenic	HLCSD, AR

* Indicates that the mutation is not included in the database. The reference database version is Human Genome 38 (hg38/GRCh38). The table is based on the guidelines of the American Society for Medical Genetics and Genomics (ACMG) and recommendations on the application of the guidelines, and the sites associated with phenotype and high pathogenic potential are selected and reported after genetic pattern, age of onset, population frequency, and hazard prediction filtering.

## 3 Discussion

HCLS facilitates the covalent binding of biotin to an inactive apocarboxylase through its carboxyl group, thereby forming the active holoenzyme (León-Del-Río et al., 2017). HCLS deficiency is a rare autosomal recessive metabolic disorder that impairs gluconeogenesis, fatty acid metabolism, and amino acid catabolism, resulting in dermatological, metabolic, respiratory, and neurological abnormalities. The age at which HCLS deficiency presents varies, with over half of the cases occurring in newborns (Wiltink et al., 2016). In this study, patients initially presented with respiratory symptoms as the primary clinical manifestation, accompanied by metabolic acidosis, dysregulation of blood sugar, and other symptoms. Specific symptoms were not initially evident, leading to delayed diagnosis and treatment. However, once the diagnosis was confirmed through genetic testing, prompt biotin therapy was administered, resulting in the correction of lactic acidosis, alleviation of symptoms, and absence of subsequent complications.

Functional studies suggest that the HCLS gene variant exon6: c.1088T>A: p.V363D is associated with impaired gene function (Dupuis et al., 1999). This variant has been observed in compound heterozygosity with the c.2434C>T variant in multiple patients. Homozygous variants at this site have also been detected in some patients (Wu et al., 2020). Regarding the HLCS: NM_001352514: exon11: c.2434C>T: p.R812* variant, it has been found to be compound heterozygous with the c.1088T>A variant. Literature reports indicate the presence of an unknown phase pathogenic variant in one patient (Tangeraas et al., 2020). This variant is located in the last exon of the gene and is predicted to undergo Nonsense-Mediated mRNA Decay (NMD) in less than 10% of the protein region. This variant is classified as “Pathogenic” in the ClinVar database and as “DM” in the HGMD (Suzuki et al., 2005; Tabor et al., 2014; Xiong et al., 2015).

Mutations in the HLCS gene (OMIM:609018) lead to HLCSD (OMIM:253270). The main manifestations of HLCSD include metabolic acidosis, global developmental delay, increased muscle tone, hyperventilation, coma, hair loss, organic aciduria, vomiting, seizures, decreased muscle tone, generalized hypotonia, irritability, elevated levels of 3-methylcrotonylglycine in urine, 3-hydroxyisovaleric aciduria, difficulty with infant feeding, hyperammonemia, rash, tachypnea, thrombocytopenia, neonatal onset, and lactic acidosis. This case report emphasizes the respiratory symptoms as the initial manifestation, which is rare and lacks specificity, making it susceptible to misdiagnosis. The genetic testing results presented in this report will undoubtedly aid in the diagnosis and treatment of HLCS deficiency in China.

The limitations of this case report include the focus on detecting SNVs within the entire exome region and a 5 bp range of splice junctions, as well as InDels within 50 bp of the exonic region. This detection method does not consider poly structures, tandem repeat sequences, GC-rich regions, and homologous similar sequences (pseudogenes). Additionally, InDels exceeding 50 bp have certain limitations. The current whole exome sequencing, due to technical constraints of whole exome capture, cannot ensure complete coverage of the exonic region. Furthermore, there is a possibility of maternal contamination and low-level mosaicism that cannot be ruled out. In addition to the known pathogenic variant c.1088T>A: p.V363D, the variant c.2434C>T: p.R812* is likely to be a contributing factor in disease development. However, further studies are required to validate these findings.

## 4 Conclusion

HLCSD is a rare autosomal recessive genetic disease resulting from mutations in the HLCS gene. The lack of specific symptoms makes it susceptible to misdiagnosis and underdiagnosis by clinicians. We present a case of HLCSD in a patient who primarily presented with respiratory symptoms. Therefore, obtaining a comprehensive medical history and performing appropriate examinations is essential in infants and young infant with unexplained respiratory disorders characterized by rapid, deep breathing. Early diagnosis and prompt treatment can mitigate the disability and mortality associated with HLCSD.

## Data Availability

The original contributions presented in the study are included in the article/supplementary material, further inquiries can be directed to the corresponding author.
